# Effects of Virtual Environmental Cues on Quiet Stance in Young Adults

**DOI:** 10.1177/00187208261420661

**Published:** 2026-02-03

**Authors:** Aliza Maqsood, Lisa K. Lavalle, Taylor W. Cleworth

**Affiliations:** 1 7991 York University, Canada; 2 4257 Queen’s University, Canada

**Keywords:** posture, kinetics, kinematics, vision, virtual environments

## Abstract

**Objective:**

The aim of this study was to explore the effect of contrast and spatial frequency intensities in a virtual environment on quiet, upright stance.

**Background:**

Visual feedback provides crucial sensory information to maintain postural control. Changes to contrast sensitivity and spatial frequency in the environment have been shown to influence postural stability; however, there is currently no work examining the influence of environmental contrast and spatial frequency on balance among young healthy populations.

**Methodology:**

28 healthy participants stood on a force plate, feet together, while wearing a head-mounted display. Participants viewed a virtual room and were exposed to four 60s conditions, each with a modified level of contrast (low or high) and spatial frequency (low or high) of the surrounding wallpaper. Center of pressure and head displacement root mean square and mean power frequency were calculated to quantify balance behavior.

**Results:**

Higher contrast reduced sway, particularly along the AP axis and on a foam surface (COP AP RMS foam: 7.56 ± 1.92 mm vs. 8.61 ± 1.70 mm; HMD AP RMS: 7.46 ± 2.57 mm vs. 8.92 ± 3.20 mm, mean ± SD). Spatial frequency affected only COP ML RMS on foam, with lower spatial frequencies producing slightly greater sway amplitude (7.93 ± 1.93 mm vs. 7.42 ± 1.75 mm).

**Conclusion:**

In conclusion, the level of both contrast and spatial frequency in the surrounding environment impact balance control during quiet, upright stance.

**Application:**

This study suggests visual environmental cues should be considered when designing environments to reduce fall risk.

## Introduction

Falls caused by balance deficits are a major concern among older adults (Lord, 2006; Shaffer & Harrison, 2007). It has been suggested that community-dwelling adults aged 65–75 years have a 25–30% likelihood of falling annually (Betrabet Gulwadi & Calkins, 2008). To maintain balance control and avoid a fall, sensory feedback is integrated from the visual, vestibular, and somatosensory systems (Peterka, 2002, 2018; Peterka & Loughlin, 2004). Specifically, the visual system offers critical feedback for balance control by providing information on the surrounding environment and one’s position and orientation relative to it (Fitzpatrick & McCloskey, 1994). For example, prior work has shown that increasing optic flow feedback through virtual reality (VR) can improve static balance control (Lavalle & Cleworth, 2023). Additionally, when the visual system is impaired through reduced visual acuity, individuals have been shown to demonstrate poorer static and dynamic balance control (Zarei et al., 2023).

Balance control can also be impacted through the visual cues that are presented in the environment (Lord, 2006). For example, previous work has shown that fall risk can be impacted by several environmental factors, such as visual contrast and spatial organization among surrounding surfaces (Betrabet Gulwadi & Calkins, 2008). Visual contrast is described as the relative difference in brightness between adjacent areas (Campbell & Maffei, 1974). Therefore, contrast sensitivity refers to the ability of the eye to discriminate between colors or shades (Campbell & Maffei, 1974). Contrast plays a crucial role in object perception, where an increase in target contrast has been shown to increase both object fixation and detection (‘t Hart et al., 2013), as well as motion detection (Edwards et al., 1996). Spatial frequency refers to how often light and dark patterns repeat over a given area, describing the level of visual detail in an image (Campbell & Maffei, 1974). High spatial frequencies correspond to fine textures and sharp edges, whereas low spatial frequencies represent broader, smoother patterns. As opposed to contrast, lower levels of spatial frequency have been shown to improve motion detection due to a reduction in interference from high-resolution details (Shi et al., 2020). Additionally, naturally occurring visual patterns in the environment often consist of lower frequency components (Shi et al., 2020), and it is also known that primates have more neurons tuned to low, compared to high frequencies (Perrone & Thiele, 2001). It is also important to note that contrast and spatial frequency are not independent visual elements, but rather depend on the level of the other to be seen; this is demonstrated through the contrast sensitivity function (Campbell & Robson, 1968). For example, if objects in a visual field contain a very low spatial frequency, higher contrast would be needed to correctly identify them.

Previous work has studied how changes in visual characteristics such as contrast and spatial frequency influence postural control, providing insights into the visual contributions to balance. Although much of the preexisting studies have emphasized older adults, studying healthy individuals can provide a baseline for interpreting age-related changes. For example, when standing quietly with eyes open, viewing targets with modified levels of spatial frequency, participants showed postural sway amplitude was greater when viewing high (8 cyc/deg) compared to low (2.5 cyc/deg) spatial frequency (Anand et al., 2003). This suggests that environments with higher levels of spatial frequency may place greater demand on postural control, potentially because of reduced reliance on global motion cues. More recent work has also shown that reductions in contrast sensitivity are associated with increased postural sway and reduced gait performance (Thompson et al., 2023), highlighting the importance of visual clarity in balance regulation. Investigating these visual parameters in younger adults offers an opportunity to characterize how spatial frequency and contrast contribute to postural control. This provides a baseline for understanding how these mechanisms may change with age or visual impairment.

Although there is previous work that has established relationships with either contrast or spatial frequency on measures of balance control, further research is still needed to understand the combined impact of modifying multiple environmental cues on postural stability, particularly among healthy adults. Studying younger participants minimizes the confounding effects of age-related sensory or motor decline and establishes a normative baseline for how visual environmental cues affect postural control (Stoffregen et al., 2007). This baseline can then inform future research examining whether these visual influences differ or intensify among older adults.

VR technology provides a promising way to study this relationship. By using a head-mounted display (HMD), participants are immersed within a computer-generated stereoscopic virtual environment. One of the benefits of using VR technology is the ability to alter the environment in a controlled setting, while still maintaining a high level of ecological validity (Assländer & Streuber, 2020). The VR-based quiet stance paradigm has been shown to elicit postural responses comparable to those observed in real-world environments, supporting its suitability for studying balance control (Assländer & Streuber, 2020). The visual scene in this study contained no moving elements other than the participant-driven optic-flow, minimizing the likelihood of visually induced motion sickness. Previous work indicates that such static or minimally dynamic virtual environments produce minimal simulator sickness in healthy young adults (Stanney et al., 2020; Weech et al., 2019). As a result, participants’ sway responses are interpreted as reflective of balance control mechanisms under controlled visual conditions. Quiet standing tasks provide a controlled way to isolate sensory and biomechanical contributions to postural control before examining more complex dynamic movements. Quiet stance is also known to minimize voluntary movement, which allows precise assessment of how visual and somatosensory inputs shape fundamental balance mechanisms (Winter, 1995; Peterka, 2002; Assländer & Peterka, 2014). Establishing these relationships under static conditions is essential for predicting how similar visual changes might affect functional balance and fall risk.

VR-based paradigms can reliably reproduce balance responses observed in real environments and allow precise manipulation of visual cues that are otherwise difficult to isolate (Ketterer et al., 2024). Virtual environments have been successfully used to investigate how motion, visual feedback, and sensory conflict influence postural stability. This supports the use of VR as a tool to examine the effects of contrast and spatial frequency on balance control. Therefore, the primary objective of this study was to use VR to understand the effect of contrast and spatial frequency on postural stability during quiet, upright stance among healthy young adults. It was hypothesized that when exposed to a virtual environment with a high level of contrast and a low level of spatial frequency, participants would demonstrate improved balance performance, as measured by decreased amplitude and increased frequency of postural measures.

## Methods

### Participants

Twenty-eight healthy young adults (mean age ± SD: 21.96 ± 1.62 years, 16 female) volunteered to participate in this study from York University and the surrounding community. Individuals reported no known neurological or musculoskeletal deficits and were required to have self-reported normal or corrected to normal vision to participate. This research complied with the tenets of the Declaration of Helsinki and was approved by the Institutional Review Board at York University. Informed consent was obtained from each participant.

### Virtual Environment

**Figure 1.**
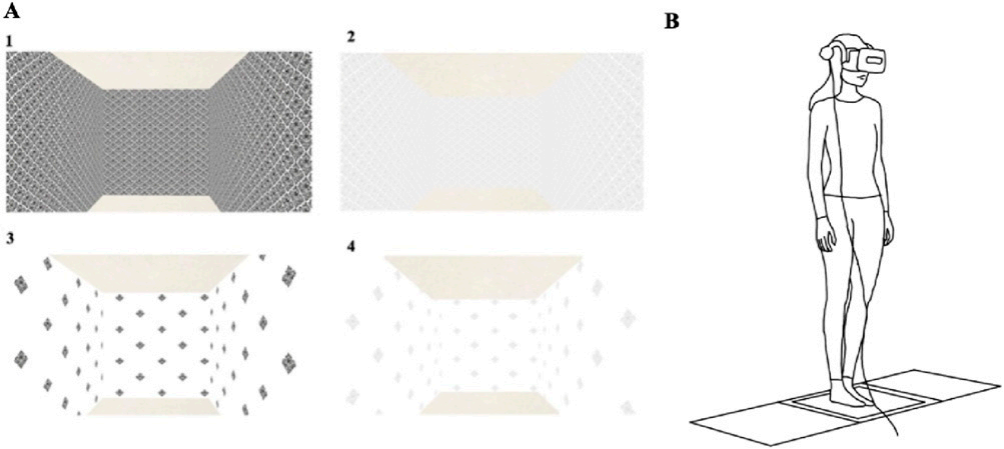
(a) Participants’ view of the VR scene is displayed for each of the four conditions: (1) high contrast and high spatial frequency, (2) low contrast and high spatial frequency, (3) high contrast and low spatial frequency, and (4) low contrast and low spatial frequency. (b) Experimental setup illustrating participants’ stance on the force plate while wearing the VR HMD.

The VR HMD used for this study had approximately a 120° horizontal field of view (HTC Vive Pro 2, HTC Corporation). The HMD provides real-time positional and rotational tracking of the user’s head via dual infrared base stations operating at 90 Hz. The visual display was updated continuously in response to participants’ natural head movements, ensuring that the optic flow remained aligned with participants’ postural sway. This configuration represents a closed-loop display, in which visual feedback dynamically reflects the participant’s movements, allowing interpretation of postural responses in terms of visual control of stance (Bardy et al., 1999; Stoffregen et al., 2000a, 2000b). The HMD’s vertical and horizontal fields of view were narrower than those of natural vision, though this difference is not expected to affect quiet stance measures (Assländer & Peterka, 2014; Schedler et al., 2024). A custom virtual environment was developed using Vizard python programming which displayed a room with four walls, a ceiling, and a floor (4 m × 4 m x 4 m; Figure 1(a)) (WorldViz, USA). The virtual position in the scene was fixed at the center of the room, where participants stood at a position equidistant from all surrounding walls. This configuration maintained a consistent visual angle across all conditions, preventing any variations in perceived distance (Bardy et al., 1999; Stoffregen et al., 2000). To modify visual environmental cues, the contrast and/or spatial frequency of the pattern on the walls was altered, excluding the ceiling and floor which remained neutral. Spatial frequency was manipulated by increasing the repetition of black patterns across the white background, enhancing the density of visual elements. Contrast was adjusted using Windows PowerPoint’s built-in image modification tools to amplify the difference between light and dark regions (Figure 1(a)). All images were manipulated prior to the experiment and rendered into the virtual scene. The wallpaper was selected to reflect patterns of complex visual environments that older adults may encounter in real life, such as decorated interiors especially in assisted care facilities (Betrabet Gulwadi & Calkins, 2008). Across trials, contrast and spatial frequency were modified generating four scenes with high and low levels of each variable: (1) high contrast, high spatial frequency, (2) low contrast, high spatial frequency, (3) high contrast, low spatial frequency, and (4) low contrast, low spatial frequency (Figure 1(a)).

In real-world settings, changes in optic flow can result from the amount of surface detail on the scene, or the observer’s distance from it. For example, a wall faraway with intricate details can produce a similar optic flow density to that of a nearby sparsely textured surface (Lestienne et al., 1977; Stoffregen et al., 2000). In this study, spatial frequency and contrast were the only manipulated variables, and to isolate their effects, the viewing distance between the participant and virtual scene was kept constant across all conditions.

### Experimental Protocol

For each trial, participants were asked to stand quietly on a force plate for 60 s with their arms resting naturally at their sides and feet placed together, while wearing the HMD (Figure 1(b)). There were eight experimental trials in total which included two surface conditions (firm and foam), each with four visual conditions. No trials were repeated. Two surface conditions were chosen as standing on a compliant surface reduces somatosensory feedback from the lower limb, while increasing the reliance on vision and vestibular feedback for balance (Schut et al., 2017). For foam conditions, participants stood on a foam pad (dimensions: 10 x 61 x 46 cm; density: 25 kg/m^3^) placed over top of the force plate. The order of the conditions was randomized across all participants to mitigate the impact of learning and fatigue. At the beginning of each trial, participants were asked to fixate their gaze on a black square shown on the wall in front of them. The black square was then removed, and participants were instructed to remain focused on the same approximate point (looking straight ahead) within the virtual environment for the duration of each trial. Between each trial, participants were given a 60 s resting period.

### Outcome Measures

Ground reaction forces and moments were sampled at 100 Hz from a single force plate (AMTI, USA). Center of pressure (COP) displacements were then calculated in MATLAB (R2023b, Mathworks Inc, USA) in the anteroposterior (AP) and mediolateral (ML) directions. The COP data was low pass filtered with a 5 Hz dual pass Butterworth filter and bias was removed by subtracting the mean COP position from the recorded signal for each trial. Head position (HeadPos) was collected in the AP direction using the HMD. The HeadPos data was collected through Vizard, converted to an analog signal using a digital lab interface (LabJack, Colorado, USA), sent to a data acquisition board (Power1401, Cambridge Electronic Design (CED), UK), and recorded at 100 Hz (Spike2, CED, UK). The HeadPos data was low pass filtered with a 5 Hz dual pass Butterworth filter and bias was removed by subtracting the mean HeadPos from the recorded signal for each trial. Root Mean Square (RMS), a measure of amplitude, and Mean Power Frequency (MPF), a measure of frequency, were calculated in MATLAB for both AP and ML COP, as well as AP HeadPos. MPF was calculated using the following formula:

$$MPF=\frac{\sum fxP\left(f\right)}{\sum P\left(f\right)}$$

where f represents the frequency of each signal and P*(f)* indicates the power at each respective frequency. This measure reflects how rapidly corrective adjustments occur, with higher MPF values indicating more rapid neuromuscular responses and greater control stiffness (Carpenter et al., 2001; Duarte & Zatsiorsky, 2000; Ruhe et al., 2011).

For the time series analysis, RMS was calculated using the following formula:

$$RMS=\sqrt{\frac{1}{n}\;\overset{n}{\underset{i=1\;}{\sum }}x_{i}^{2}}$$

where x represents the data sample and n indicated the number of data points.

Including both RMS and MPF allows assessment of not only the magnitude but also the temporal organization of sway, offering a better understanding of balance behavior. These metrics were used to quantify spatial aspects of postural control and to capture how modified visual information influences sway magnitude (Peterka, 2002; Prieto et al., 1996).

### Statistical Analysis

A 2 (low and high contrast) x 2 (low and high spatial frequency) repeated measures analysis of variance (ANOVA) using SPSS (IBM Corp, N.Y., USA) was completed for each outcome measure. For each outcome measure, independent ANOVAs were run separately for firm and foam surfaces since the effect of surface condition on quiet stance is already well-known (Lavalle & Cleworth, 2023; Schut et al., 2017). To assess normality across all variables, Shapiro–Wilk tests and histograms were used. Z-scores were calculated to identify outliers, with scores of ±3 indicating an outlier. All outlying values were then adjusted to 3 standard deviations from the mean (Field, 2009). Despite these adjustments, the data still did not conform to a normal distribution; however, due to the robust nature of repeated measures ANOVA and the central limit theorem approximating normality, no corrections for normality were used for analysis (Field, 2009). The statistical significance was set at an α-level of 0.05 and Sidak corrections were used to account for multiple comparisons.

## Results

### Amplitude (RMS)

**Figure 2.**
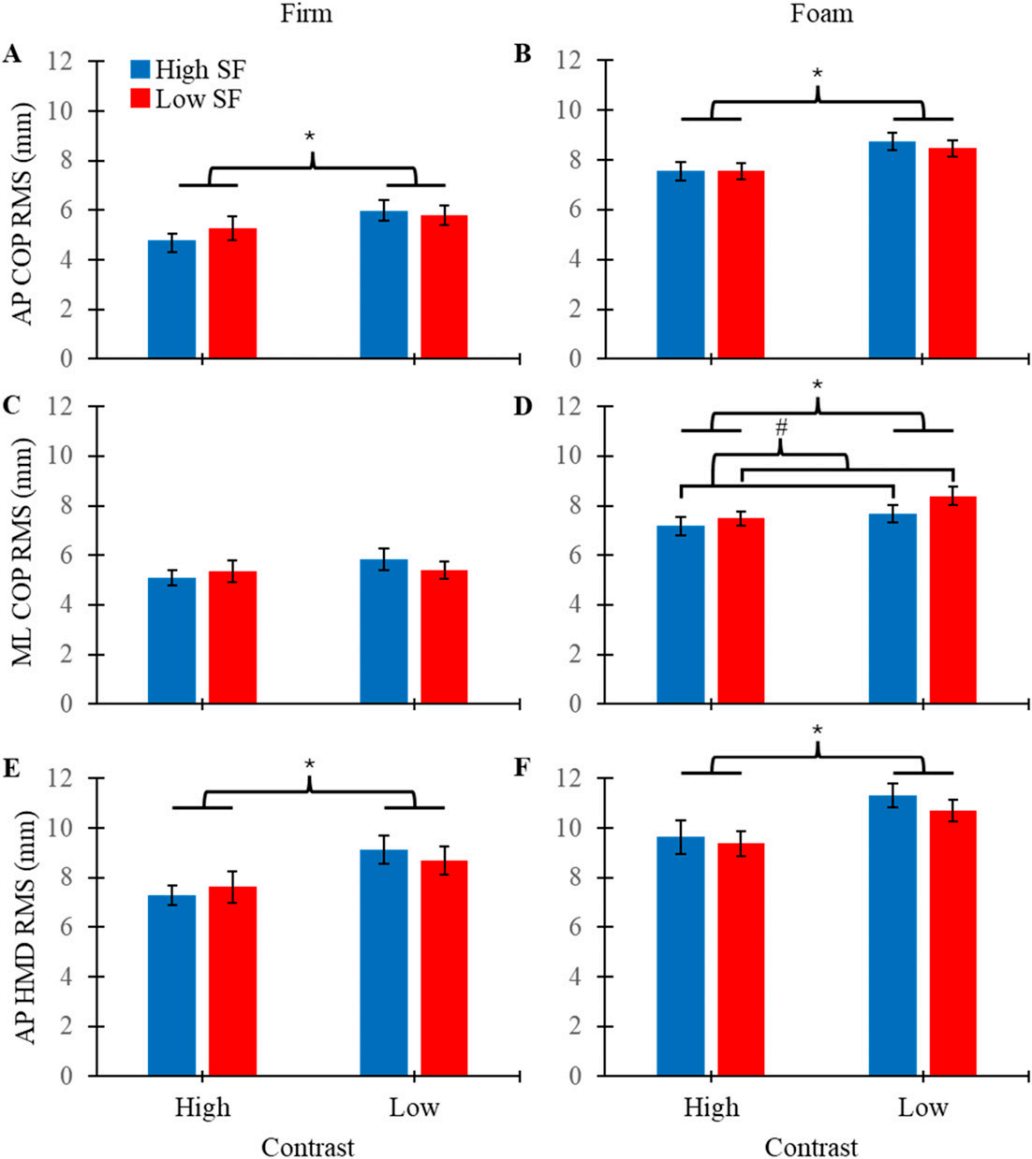
Mean RMS values (+/− SEM) plotted for AP COP, ML COP, and AP HeadPos for each contrast level while standing on firm and foam surfaces. Blue bars represent high spatial frequency and red bars represent low spatial frequency. Significance brackets are used to denote significant main effects of contrast (*) and spatial frequency (#).

There were significant main effects of contrast on AP COP RMS and AP HeadPos RMS for both surface conditions, while no significant interaction effects were observed (Table 1). AP COP and AP HeadPos RMS increased during low contrast compared to high contrast conditions (Figure 2(a, b, e and f)). There were no main effects or interaction effects for ML COP RMS for firm surface conditions. However, there was a significant main effect of spatial frequency on ML COP RMS while standing on foam, with no significant interaction effect observed (Table 1). ML COP RMS increased during low compared to high spatial frequency conditions while standing on foam (Figure 2(d)). No other significant main effects or interaction effects were observed for RMS data.

**Table 1.** Summary of Repeated Measures ANOVA Results for RMS and MPF Measures Across Both Firm and Foam Surface Conditions

		Contrast			Spatial frequency			Contrast * spatial frequency		
		F (df 1, 27)	*p* value	Partial η2	F (df 1, 27)	*p* value	Partial η2	F (df 1, 27)	*p* value	Partial η2
AP COP	RMS firm	**6.122**	**0.020**	0.19	0.291	0.594	0.01	1.491	0.233	0.05
	RMS foam	**17.12**	**< 0 .001**	0.39	0.447	0.510	0.02	0.388	0.538	0.01
	MPF firm	1.919	0.177	0.07	1.790	0.192	0.06	0.525	0.475	0.02
	MPF foam	0.000	0.983	0.00	0.830	0.370	0.03	0.099	0.755	0.00
ML COP	RMS firm	0.864	0.361	0.03	0.095	0.760	0.00	2.078	0.161	0.07
	RMS foam	**10.62**	**0.003**	0.28	**6.125**	**0.020**	0.19	0.792	0.381	0.03
	MPF firm	0.005	0.943	0.09	2.515	0.124	0.09	0.161	0.692	0.01
	MPF foam	0.517	0.478	0.02	0.000	0.990	0.00	0.112	0.741	0.00
AP HMD	RMS firm	**8.674**	**0.007**	0.24	0.012	0.912	0.00	0.833	0.833	0.03
	RMS foam	**10.43**	**0.003**	0.28	1.267	0.270	0.05	0.236	0.631	0.01
	MPF firm	1.418	0.244	0.05	1.236	0.276	0.04	0.110	0.110	0.00
	MPF foam	0.124	0.727	0.12	2.869	0.102	0.09	3.661	0.066	0.12

Bolded *p* values indicate statistical significance.

### Frequency (MPF)

**Figure 3.**
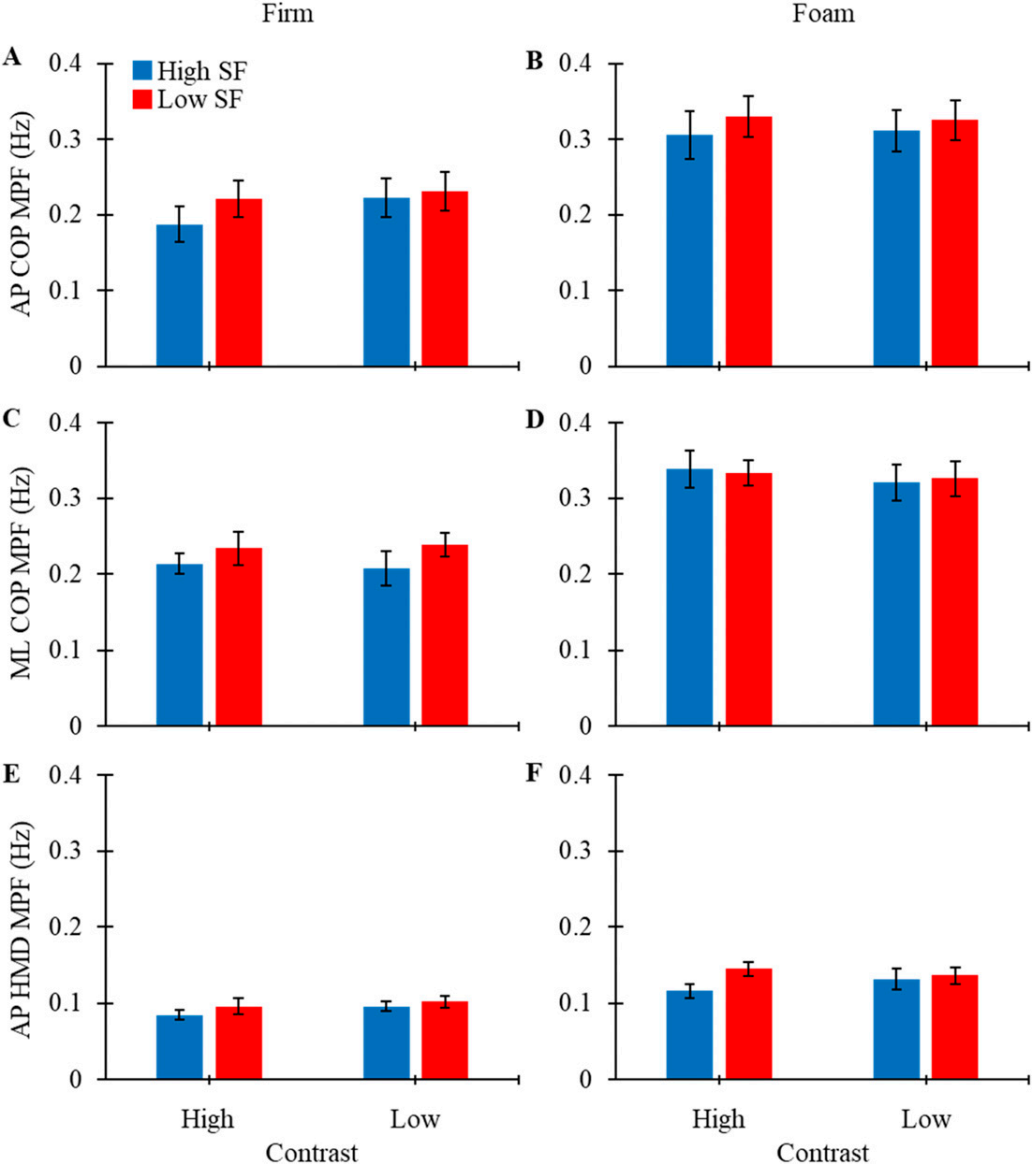
Mean MPF values (+/− SEM) plotted for AP COP, ML COP, and AP HeadPos for each contrast level while standing on firm and foam surfaces. Blue bars represent high spatial frequency and red bars represent low spatial frequency.

No significant main effects of either contrast or spatial frequency, or significant interaction effects were observed for any MPF-related outcome measures (Table 1; Figure 3). There was a trend towards a significant interaction effect for AP HeadPos MPF, where AP HeadPos MPF was larger in the low SF compared to high SF when contrast was low (F_(1, 27)_ = 3.661, p = 0.066).

## Discussion

The aim of this study was to understand the contribution of combined visual environmental cues to balance control during quiet stance among healthy adults. Specifically, the effects of modified contrast and spatial frequency levels on quiet, upright stance were examined while participants were exposed to a virtual environment. Overall, the results demonstrated an inverse relationship between contrast and balance-related movement amplitude, where a decrease in contrast led to increased amplitude of COP and HeadPos in the AP direction, independent of surface condition. Additionally, spatial frequency had an effect on COP ML-related movement on foam surface conditions only, where balance-related movement amplitude increased with low spatial frequency. The current results, derived from healthy younger adults, highlight how visual contrast and spatial frequency influence postural control when sensory and motor systems are fully functional. It is likely that older adults, who typically exhibit reduced contrast sensitivity and slower sensory integration (Owsley, 2011; Shaffer & Harrison, 2007), would show amplified responses to similar visual manipulations. Thus, the present findings provide a foundation for predicting how age-related visual declines might further destabilize postural control.

The measures of RMS amplitude decreased when observing high contrast environments. It is possible that the higher contrast conditions provided clearer visual feedback as compared to the lower contrast conditions (Lord & Menz, 2000) and it is well known that increased optic flow feedback enhances postural stability (Lavalle & Cleworth, 2023). These findings suggest that postural control can be selectively tuned to visual stimuli based on environment and task demands (Balasubramaniam et al., 2000; Stoffregen et al., 2000). In this study, a stationary black square was presented briefly at the start of each trial as a focal point reference, after which participants viewed only the textured background. Therefore, participants’ postural responses were driven by the optic flow of the whole background scene, rather than by attention to a specific feature. Previous work has found that high contrast environments improve visual feedback by increasing the ability to detect objects and changes in surface, allowing individuals to perceive the environment more clearly (Figueiro et al., 2011). Through this enhanced perception of visual cues with increased contrast, stability is known to improve especially among older adults who depend more heavily on visual cues (Figueiro et al., 2011). Further, previous studies have shown that older adults with reduced contrast sensitivity, with no loss of visual acuity, demonstrate poorer postural stability and impaired mobility, which is specifically seen on foam surface conditions due to reduced somatosensory feedback (Lord & Menz, 2000; Thompson et al., 2023).

Although increased amplitude has traditionally been interpreted as diminished postural control (Peterka, 2002), recent work suggests that stability cannot be evaluated solely through spatial measures. The temporal structure and complexity of sway patterns also provide insight into how postural control evolves over time (Lin et al., 2008; Stergiou & Decker, 2011). Therefore, this study’s findings should be viewed as describing changes in the spatial characteristics of sway under different visual conditions, rather than as comprehensive indicators of overall stability.

Spatial frequency may have a larger role in maintaining balance control while individuals are largely reliant on visual feedback, as spatial frequency only impacted quiet stance while standing on foam. Standing on a compliant surface is known to influence the sensory contributions to postural control, ultimately increasing the visual contributions to controlling upright stance (Schut et al., 2017). Conditions that modify sensory contributions to balance have important implications for populations such as older adults or those with vestibular or somatosensory deficits who increase reliance on the visual system to provide feedback for balance control (Manchester et al., 1989; Yeh et al., 2014). While on foam, the results of this study showed that COP amplitude decreased with high spatial frequency, which is contrary to both our hypothesis and work by Anand et al., which showed that postural sway was reduced with low spatial frequency (Anand et al., 2003). One explanation for this discrepancy is that the visual targets used by Anand et al. had a surface area of approximately 1 m^2^, while this study manipulated visual feedback throughout the entire visual field in VR. Further, our results also suggest that high spatial frequency had a stabilizing effect on posture, specifically while the environment was low in contrast (Figure 2). It is therefore possible that higher levels of spatial frequency are particularly important in environments of low contrast, which would otherwise lack necessary visual cues needed to maintain balance. Previous work has also shown that there are ideal levels of spatial frequencies (0.5–1.0 cyc/deg), as higher or lower levels increase postural sway (Kunkel et al., 1998). However, these effects can change. When the environment has lower levels of contrast, higher levels of spatial frequency (2.0–4.0 cyc/deg) lead to reduced postural sway, as increased contrast can compensate for a lack of clear visual boundaries to ensure that the visual system receives adequate input to stabilize posture (Anand et al., 2003). Regardless, high spatial frequencies can provide finer visual details, reduce COP amplitude, and generate stabilizing effects in certain situations. Lastly, previous work has shown that image stabilization is maintained at retinal slip velocities below 2–4°/s, which can be achieved through reduced head translations (Crane & Demer, 1998; Demer et al., 1994). With these findings, it is reasonable to suggest that increased spatial frequency, and therefore increased optic flow feedback, could stabilize head amplitude through reducing both AP and ML translations; however, further work would be needed to investigate this hypothesis.

Although this study interpreted the effects of the foam surface primarily in terms of sensory re-weighting, the manipulation also has important biomechanical implications. Standing on a compliant surface alters the mechanical properties of the base of support and changes the motor strategies required to maintain equilibrium. Greater compliance reduces the efficiency of ankle torque transmission and increases the magnitude of joint rotation needed to displace the center of mass (Horak & Nashner, 1986; Peterka, 2002). Thus, differences in sway between firm and foam surfaces may arise not only from changes in sensory feedback reliability but also from biomechanical adjustments inherent to maintaining stability on a compliant substrate. Even if sensory perception were perfectly accurate, the foam condition would still be expected to yield distinct postural patterns due to these altered mechanical constraints. Consequently, the larger sway observed under low contrast conditions on foam likely reflects an interaction between increased visual uncertainty and the additional mechanical demands of controlling posture on a compliant surface.

Contrary to our hypothesis, neither contrast nor spatial frequency significantly impacted the MPF of COP or HeadPos in this study. However, these findings are specific to healthy young adults in a controlled virtual environment. Although VR and real-world scenes produce similar postural responses during quiet stance (Assländer & Streuber, 2020), it is possible that older adults, who experience age-related sensory declines, or individuals exposed to more visually complex real-world environments might exhibit different frequency responses. Previous work has shown that increasing optic flow feedback leads to increased frequency of COP (Lavalle & Cleworth, 2023). Changes to COP frequency are largely driven through corrective torque, generating ankle-stabilizing moments to maintain the COP within the base of support (Fitzpatrick et al., 1996; Peterka, 2018). Previous work has also shown that when balance tasks become more challenging through altering sensory feedback, corrective postural adjustments occur at more proximal joints, such as the hip (Riemann et al., 2003). Given that this study modified visual feedback, it is therefore possible that participants utilized alternative postural strategies, such as stiffening their trunk. This could have contributed to changes in frequency not being captured by COP outcome measures. Further kinematic analyses would be needed to assess changes to full body kinematics to better understand the relationship between visual feedback modifications, full body postural strategies, and their impact on postural stability.

### Limitations and Future Research

Due to technical constraints, head kinematics could only be captured in the AP direction. This meant that ML head position could only be estimated using head displacements captured in the AP direction. Given ML COP RMS was influenced by spatial frequency, it is unclear whether ML HeadPos RMS would have exhibited similar results with modified spatial frequency. A second limitation in the current study is the characterization of high and low contrast/spatial frequency as relative measures. For example, high spatial frequency conditions had more light and dark images in the visual field compared to low spatial frequency conditions. However, the purpose of this study was to observe the impact on modifying these variables on balance control, rather than quantifying the impact of absolute values. While interpreting the results of this study, it is important to note that contrast and spatial frequency follow the contrast sensitivity function, where increasing these visual cues to a maximal degree could actually decrease the availability of visual feedback (Robson, 1966). Therefore, further work is still needed to quantify the level of contrast and spatial frequency in an environment that would optimize postural control.

Next, there was no nonlinear, or full body kinematic measures of sway (e.g., sample entropy) included in the analysis of this study. Future work could examine whether variations in contrast or spatial frequency also affect the nonlinear dynamics of postural control.

A final limitation concerns potential differences in visual acuity or retinal blur across participants. Previous research has shown that postural responses to optic flow remain relatively stable across variations in focus (Anand et al., 2003), which suggests that moderate blur does not eliminate the visual control of stance. Both contrast and spatial frequency perception are affected by visual clarity, therefore small corrections in visual acuity could have contributed to individual difference in postural responses. Participants reported normal-to-corrected vision on the screening questionnaire, but no objective vision testing was performed. Thus, future studies should include standardized visual assessments to limit this potential confound.

## Conclusions

In conclusion, the overall findings of this study demonstrate that manipulating contrast and spatial frequency in VR influences balance control during quiet stance among healthy adults. Higher levels of contrast decreased movement amplitude during upright stance, whereas higher levels of spatial frequency decreased ML movement amplitude, specifically while standing on foam. Further, participants were overall more sensitive to changes in contrast and spatial frequency in the environment during conditions of decreased somatosensory feedback. Therefore, although both contrast and spatial frequency are important for postural stability, contrast has a greater impact on the amplitude of movement compared to spatial frequency. Understanding the influence of visual environmental factors on balance control can help us to design safer environments for populations who are vulnerable to balance deficits, such as older adults who are particularly reliant on vision for balance. For example, by optimizing the contrast and spatial frequency in visual environments, it may be possible to minimize fall risk. Future research could apply this study protocol to patients with balance deficits to enhance our understanding on the relationship between visual cues and quiet stance among clinical populations.

## Key Points

• Modifying visual environmental cues in virtual reality impacted quiet stance on both firm and foam surfaces.
• Increased contrast in virtual environments decreased the amplitude of anteroposterior and mediolateral (ML) postural sway, while increasing spatial frequency reduced the amplitude of ML sway while standing on foam.
• Optimizing the visual environmental design of residential spaces could help improve postural control among populations with increased fall risk.
